# Impact of contact tracing on COVID-19 mortality: An impact evaluation using surveillance data from Colombia

**DOI:** 10.1371/journal.pone.0246987

**Published:** 2021-03-04

**Authors:** Andres I. Vecino-Ortiz, Juliana Villanueva Congote, Silvana Zapata Bedoya, Zulma M. Cucunuba

**Affiliations:** 1 Department of International Health, Johns Hopkins Bloomberg School of Public Health, Baltimore, MD, United States of America; 2 Research Office, San Ignacio University Hospital, Bogota, Colombia; 3 Planning Department, Government of the Department of Antioquia in Colombia, Medellín, Colombia; 4 MRC Centre for Global Infectious Disease Analysis, Imperial College London, London, United Kingdom; 5 Department of Clinical Epidemiology and Biostatistics, School of Medicine, Pontificia Universidad Javeriana, Bogota, Colombia; Universidad Adolfo Ibanez, CHILE

## Abstract

**Background:**

Contact tracing is a crucial part of the public health surveillance toolkit. However, it is labor-intensive and costly to carry it out. Some countries have faced challenges implementing contact tracing, and no impact evaluations using empirical data have assessed its impact on COVID-19 mortality. This study assesses the impact of contact tracing in a middle-income country, providing data to support the expansion and optimization of contact tracing strategies to improve infection control.

**Methods:**

We obtained publicly available data on all confirmed COVID-19 cases in Colombia between March 2 and June 16, 2020. (N = 54,931 cases over 135 days of observation). As suggested by WHO guidelines, we proxied contact tracing performance as the proportion of cases identified through contact tracing out of all cases identified. We calculated the daily proportion of cases identified through contact tracing across 37 geographical units (32 departments and five districts). Further, we used a sequential log-log fixed-effects model to estimate the 21-days, 28-days, 42-days, and 56-days lagged impact of the proportion of cases identified through contact tracing on daily COVID-19 mortality. Both the proportion of cases identified through contact tracing and the daily number of COVID-19 deaths are smoothed using 7-day moving averages. Models control for the prevalence of active cases, second-degree polynomials, and mobility indices. Robustness checks to include supply-side variables were performed.

**Results:**

We found that a 10 percent increase in the proportion of cases identified through contact tracing is related to COVID-19 mortality reductions between 0.8% and 3.4%. Our models explain between 47%-70% of the variance in mortality. Results are robust to changes of specification and inclusion of supply-side variables.

**Conclusion:**

Contact tracing is instrumental in containing infectious diseases. Its prioritization as a surveillance strategy will substantially impact reducing deaths while minimizing the impact on the fragile economic systems of lower and middle-income countries. This study provides lessons for other LMIC.

## Introduction

Since the first case report on December 31, 2019 [1], the rapid spread of the novel coronavirus (SARS CoV-2) led to the worst pandemic in the century [2]. In April 2020, over 2.4 million cases were detected, and up to 165,000 deaths were reported worldwide. By mid-July, more than 14 million cases and 600,000 deaths were reported worldwide [3], being the region of the Americas the most affected in the world with more than half of all cases. Specifically, the Latin American region has been hard hit by the COVID-19 epidemic due to the confluence of infectious disease dynamics, health, and socioeconomic factors. Poverty, homelessness and crowded housing conditions, a prevalent informal economy, inequality, high prevalences of noncommunicables diseases (NCD), and weak health and social service networks have collided, being the hardest hit region in the world [4–6].

For this reason, the main challenge for all countries, but particularly for middle and lower-income ones (LMIC), is to contain the spread of the virus while minimizing the adverse consequences of lockdowns and other measures that threaten recent gains in terms of poverty reduction [7]. This fact has led to calls for better instruments to strengthen epidemiological surveillance tools to break transmission chains and reduce the adverse consequences of both COVID-19 and non-pharmaceutical interventions [8]. A key tool to break transmission chains is contact tracing [9], which has proved its effectiveness in controlling other deadly diseases such as the 2014 Ebola outbreak in West Africa [9, 10].

In the specific case of COVID-19, many different strategies have emerged to improve contact tracing, ranging from Northern Ireland that has performed contact tracing through telephone calls [11] to South Korea and China, which have used mobile phone apps [12]. Previous models have suggested the high value that contact tracing can have on curbing the transmission [12–14].

However, contact tracing faces many challenges in LMICs throughout the policy cycle. Starting at the agenda-setting phase, where decision-makers might struggle to see the immediate value of this strategy compared to its logistical challenges, all the way to the implementation, monitoring, and evaluation where performance might be hard to measure [7, 15–19]. Furthermore, implementing a coordinated response across levels of government (national, subnational, etc.) in an emergency represents an additional challenge that many LMICs have struggled.

Therefore, contact tracing is likely to be left behind as a useful tool to curb outbreaks. For this reason, data on the effectiveness of contact tracing on mortality is critical, so decision-makers can make better-informed decisions on whether to include contact tracing within their priority setting processes in the management of the COVID-19 pandemic.

Colombia’s political division comprises 37 geographical units, 32 departments, and five districts (Santa Marta, Barranquilla, Buenaventura, Cartagena, and Bogota) located within those departments. In Colombia, contact tracing must be done jointly by both departmental/district and municipal governments. Departmental/district and local governments are in charge of identifying contacts, testing them, and isolating them. The departmental heterogeneity on contact tracing performance represents an opportunity to assess the differential impact of contact tracing on mortality across these departments, as these have different technical baseline capabilities to implement contact tracing. Therefore, this study aims to determine the extent to which differences in contact tracing performance might explain changes in COVID-19 mortality in Colombia. This study will become a key resource for decision makers about the effectiveness of this tool and more broadly about how epidemiological surveillance can reduce the burden of COVID-19, particularly for LMIC.

## Materials and methods

### Data

This study uses publicly available and anonymized data on all confirmed novel Coronavirus cases obtained from the "Open Data" portal of the Colombian National Institute of Health [20]. Our primary sample comprises 54,931 cases identified between March 2 and June 16, 2020 (135 days of observation period). All data for the primary sample was obtained on June 17, 2020. All novel Coronavirus cases are confirmed through Polymerase Chain Reaction in Real Time (PCR-RT). We included data for all 32 departments plus five districts for a total of 37 geographical units, as described earlier. The primary sample is used for our main analysis and the first of the two robustness checks we conduct in this study (Eqs 1 and 2 below, respectively). For the second robustness check (Eq 3 below), we took a different set of cases outside our primary sample and included the daily departmental percentage of ICU occupation. Data on ICU supply for both robustness checks was obtained from the Ministry of Health dashboard [21]. All the cases of this new sample were obtained on July 17, 2020, and correspond to cases diagnosed between June 17 and July 15 (study period of 28 days), for a total of 112,279 confirmed cases. Cases for the robustness check double the original sample due to the exponential increase in COVID-19 cases in Colombia in mid-2020.

Importantly, we assume that this percentage of detected cases vs. undetected cases is constant in the study period. Since we wanted to make sure that potentially increasing test capabilities in the study period do not introduce a temporal bias in the variable of active cases, we tested the statistics around asymptomatic cases in this period. We used asymptomatic cases because the figures on asymptomatic cases better reflect changes in testing capabilities compared to those with symptoms, as the latter end up being tested. We found no statistically significant changes in the trends of asymptomatic cases in the observation related to the deaths (p = 0.76).

### Variables

A useful performance metric to ascertain the completeness and quality of epidemiological surveillance is the proportion of cases that are identified through contact tracing out of all identified cases [22]. In Colombia, cases identified through contact tracing are called "related cases", a confirmed case detected through a PCR-RT test with a known epidemiological link [23]. This study uses as main independent variable, the logarithm of the 7-day moving average daily departmental proportion of related cases out of all cases detected, as a proxy metric for contact tracing performance. Our outcome measure is the logarithm of the 7-day moving average of the daily departmental COVID-19 confirmed deaths (N = 1,853 deaths for the primary sample; N = 3,738 deaths for the sample of the second robustness check). By obtaining logarithms for both metrics, we can interpret the results as elasticities (percentage changes).

As controls, we used:

1. data from the Google Community Mobility Reports [24] for six modes of mobility: retail/recreation activities, trips to grocery/pharmacy stores, parks, transit stations, and workplace sites. As the Google mobility report has data discriminated by the department but not by district, we imputed the mobility data of the departments where the corresponding districts are located as follows: Santa Marta in Magdalena department; Barranquilla in Atlantico department; Buenaventura in Valle del Cauca department, and Cartagena in Bolivar department. The capital district of Bogota is included in Google as a separate department. The mobility index corresponds to the percentage change in mobility compared to the baseline, which is the median value for the corresponding day of the week, during the 5-week period between Jan 3-Feb 6, 2020.
2. To capture concurrent time-varying epidemiological factors that might affect surveillance (i.e., lack of capacity to surveil beyond a given number of cases or changes in testing capacity), we calculated the number of daily active cases by department defined as the number of daily cases diagnosed by department minus those recovered or deceased. This implies that there are days in which the value might become negative if the number of deaths or recovered cases surpasses the new number of cases in a given day in any given location. For the definition of active cases, we used the date of diagnosis. In the case of 622 cases from the primary sample and 3,706 from the second robustness check sample, the date of diagnosis was not available. Therefore, in those cases we imputed the date of notification to the INS.
3. Finally, and to capture potential non-linear effects, we used the second-degree polynomial for the corresponding lagged variable of contact tracing performance as control.

### Analysis

Our empirical strategy takes advantage of the heterogeneity of both the proportion of cases that resulted from contact tracing, as a proxy of contact tracing performance for each department over a 135-days observation period, and mortality. We evaluate impact of contact tracing on COVID-19 mortality using a fixed-effects model, a widely accepted econometric technique to assess the effectiveness of public policy interventions that control for both unobservable and observable time-invariant variables that might confound that statistical relationship. With this identification approach, we prevent baseline heterogeneity across departments (e.g., institutional strength of local health departments, funding, built capacity, etc.) biasing our results. As the effect of contact tracing will likely be observed at a population level between 2 and 6 weeks after contact tracing is implemented [25], we obtained four different lags for the contact tracing variable at 21 days (model 1), 28 days (model 2), 42 days (model 3), and 56 days (model 4). We incrementally included lags to assess the effectiveness of contact tracing (sequential model).

In Eq 1, we present the full log-log fixed-effects model (model 4) used in this study:

$$\begin{array}{l} {Log(deaths)}_{it}=\sum_{d=21}^{D}\beta_{p}{Log(Percentage\;of\;cases\;traced)}_{it-d}\\ \;+\sum_{d=21}^{D}\beta_{q}{{Log(Percentage\;of\;cases\;traced)}_{it-d}}^{2}\\ \;+\sum_{d=21}^{D}{\beta_{r}(\hat{Percentage\;of\;change\;in\;mobility\;indices})}_{it-d}\\ \;+{\beta_{1}{Log(Prevalence\;of\;active\;cases)}_{it}+\mu }_{it}+{a_{i}}_{} \end{array}$$ Eq 1

Where *i* is the department, *t* represents the current day, *β*_*p*_, *β*_*q*_, *β*_*r*_, *β*_1_ represent the coefficients for the corresponding vectors for lagged and non-lagged variables, *μ* is the error term, and *a*_*i*_ represents the department-specific intercept. *d→D* represents each of the four *d* discrete lagged periods observed, that is *t* minus: 21, 28, 42, 56.

### Robustness checks

In our robustness checks, we included supply-side effects as they are likely an important determinant of COVID-19 mortality, particularly in terms of the supply of intensive care units (ICU). We used two different variables to account for these supply-side conditions 1) the logarithm of the number of total ICU beds available by department as of June 14, 2020 (time-invariant variable presented in Eq 2); and 2) the percentage of ICU occupation by day and department between June 17 and July 15 (time-varying variable presented in Eq 3). Data on both ICU beds installed and the percentage occupation of ICU beds is obtained from the Ministry of Health dashboard [21].

In consequence, to account for total available beds by department, we used a random-effects specification to introduce a time-invariant variable. In Eq 2, we present the log-log full random-effects model (Model 4) used in this study:

$$\begin{array}{l} {Log(deaths)}_{it}=\sum_{d=21}^{D}\beta_{p}{Log(Percentage\;of\;cases\;traced)}_{it-d}\\ \;+\sum_{d=21}^{D}\beta_{q}{{Log(Percentage\;of\;cases\;traced)}_{it-d}}^{2}\\ \;+\sum_{d=21}^{D}{\beta_{r}(\hat{Percentage\;of\;change\;in\;mobility\;indices})}_{it-d}\\ \;+\beta_{1}{Log(Prevalence\;of\;active\;cases)}_{it}{+{\beta_{2}{Log(ICU\;beds\;available)}_{i}+\mu }_{it}}_{}\\ \;+a_{it} \end{array}$$ Eq 2

Where *i* is the department, *t* represents the current day, *β*_*p*_, *β*_*q*_, *β*_*r*_, *β*_1_, *β*_2_ represent the coefficients for the corresponding vectors for lagged and non-lagged variables, *μ* is the error term and *a*_*it*_ represents the within-department error term. *d→D* represents each of the four *d* discrete lagged periods observed, that is *t* minus: 21, 28, 42, 56.

Moreover, to capture the mortality effects of time-varying supply-side conditions, we used a fixed-effects model to capture the time-varying effects of ICU occupation on concurrent mortality. As the available period of study was 28 days, we only ran model 1 (21 days) and 2 (28 days) on this robustness check. In Eq 3, we present the log-log fixed-effects model (Model 2) used in this study:

$$\begin{array}{l} {Log(deaths)}_{it}\\ =\sum_{d=21}^{D}\beta_{p}{Log(Percentage\;of\;cases\;traced)}_{it-d}\\ +\sum_{d=21}^{D}\beta_{q}{{Log(Percentage\;of\;cases\;traced)}_{it-d}}^{2}\\ +\sum_{d=21}^{D}{\beta_{r}(\hat{Percentage\;of\;change\;in\;mobility\;indices})}_{it-d}\\ +\beta_{1}{Log(Prevalence\;of\;active\;cases)}_{it}{+{\beta_{2}{log(Percentage\;of\;ICU\;occupation)}_{it}+\mu }_{it}}_{}+a_{i} \end{array}$$ Eq 3

Where *i* is the department, *t* represents the current day, *β*_*p*_, *β*_*q*_, *β*_*r*_, *β*_1_, *β*_2_ represent the coefficients for the corresponding vectors for lagged and non-lagged variables, *μ* is the error term and *a*_*i*_ represents the department-specific intercept. *d→D* represents each of the two *d* discrete lagged periods observed, that is *t* minus: 21, 28.

## Results

We studied 54,931 confirmed cases from March 2 to June 16. Total daily cases by department ranged between 1 to 567 cases with a mean of 26.54 cases. Regarding the number of active cases, the maximum was 608 with a mean of 15.08, ICU beds per department ranged from 1 to 1206 with a mean of 276.6. The average of cases identified through contact tracing in our sample is 30.91%, ranging between 0% to 100%. Total deaths by department by June 16 spanned between 1 to 24 with a mean of 2.65. Mobility indices averaged between -47% and -69%, with overall mobility reduced across all departments. Table 1 presents these figures and the figures for the sample for the robustness check.

**Table 1:** Descriptive statistics of the sample.

	Primary sample			Sample for robustness check		
	Mean	Minimum	Maximum	Mean	Minimum	Maximum
Total daily new cases by department	26.54	1.00	567.00	113.01	12112.00	2112.00
Number of active cases (incident cases)	15.08	-461.00	608.00	2064.56	338570.00	39238.00
Number of ICUs beds per department	276.50	1.00	1206.00		1.00	1206.00
Percentage of ICU occupation by department				56.12	0%	10000%
Percentage of cases traced by department	30.91%	0%	100%	5.53%	0%	100%
Total deaths by department	2.65	1.00	24.00	6.16	0.00	54.00
Percentage change in mobility to retail/recreation activities	-67.68	-93.00	5.00		-89.00	-24.00
Percentage change in mobility to grocery/pharmacy stores	-47.19	-91.00	32.00		-82.00	10.00
Percentage change in mobility in parks	-59.79	-91.00	10.00		-82.00	-12.00
Percentage change in mobility to transit stations	-69.25	-100.00	17.00		-100.00	-6.00
Percentage change in mobility to workplace sites	-46.96	-89.00	25.00		-79.00	7.00

Note: Primary sample is comprised by 54,931 confirmed cases reported to the National Institute of Health. Surveillance data covers the period between March 2, 2020 and June 16, 2020. Cases are defined as confirmed (PCR-RT) cases. Deaths include deaths of confirmed cases. Departments represent 32 departments plus 4 special districts (Santa Marta, Cartagena, Buenaventura and Barranquilla) and the capital district (Bogotá). Mobility data is obtained from Google and the values reflect percentage changes with regards to the baseline. the data corresponds on the mobility index in six different modes of mobility: retail/recreation activities, trips to grocery/pharmacy stores, parks, transit stations and workplace sites. The mobility index corresponds to the percentage change with regards to the baseline, which is the median value for the corresponding day of the week, during the 5-week period Jan 3-Feb 6, 2020. The number of active cases is the difference between new daily cases and recovered cases/deaths, in a given department. Therefore, it can be negative. Sample for robustness checks is comprised by 112,279 confirmed cases reported to the National Institute of Health between June 17, 2020 and July 15, 2020 for a 28-day observation period. Departments represent 26 departments plus 4 special districts (Santa Marta, Cartagena, Buenaventura and Barranquilla) and the capital district (Bogotá). We excluded the departments of Amazonas, Choco, Guaviare, Guainia, Vaupes, and Vichada because they did not have consistent data on ICU occupation in the 28-day sub study period.

As shown in Table 2, performance metrics for contact tracing in the previous 21 to 56 days significantly predict mortality reductions in Colombia. An increase in 10% in the proportion of cases identified through contact tracing is related to mortality reductions in the next 21 to 56 days of between 2.1 and 2.3%, depending on the model. Second-order polynomials reveal a slight but statistically significant convex behavior. As these results are from cases obtained before the surveillance system is overwhelmed, it is possible that as cases increases, the second derivative turns concave, and marginally decreasing returns might take place.

**Table 2:** Fixed effects design at the department level on the effect of contact tracing on COVID-19 mortality.

Log of the number of COVID-19 deaths	Model 1 (lag for 21 days)			Model 2 (lags for 21 and 28 days)			Model 3 (lags for 21, 28, and 42 days)			Model 4 (lags for 21, 28, 42, and 56 days)		
	Effect	SE	*p*	Effect	SE	*p*	Effect	SE	*p*	Effect	SE	*p*
Logarithm of the percentage of cases traced 21 days before	-0.212	0.036	<0.001	-0.210	0.038	<0.001	-0.212	0.039	<0.001	-0.190	0.040	<0.001
Logarithm of the percentage of cases traced 28 days before				-0.011	0.033	0.738	-0.001	0.034	0.981	-0.010	0.035	0.782
Logarithm of the percentage of cases traced 42 days before							-0.003	0.029	0.929	0.005	0.030	0.868
Logarithm of the percentage of cases traced 56 days before										-0.032	0.025	0.211
Squared of the logarithm of the percentage of cases traced 21 days before	0.009	0.002	0.844	0.009	0.002	<0.001	0.009	0.002	<0.001	0.008	0.002	<0.001
Squared of the logarithm of the percentage of cases traced 28 days before				0.001	0.001	0.409	0.000	0.001	0.789	0.001	0.001	0.244
Squared of the logarithm of the percentage of cases traced 42 days before							0.001	0.001	0.174	0.001	0.001	0.014
Squared of the logarithm of the percentage of cases traced 56 days before										0.001	0.001	0.056
R-square (within)	0.503			0.523			0.512			0.507		
R-square (between)	0.519			0.399			0.428			0.438		
Observations (department/day units)	549			542			521			497		

Note: N = 54,931 confirmed cases reported to the National Institute of Health. The outcome is the logarithm of the daily number of COVID-19 deaths (7-day moving average) mortality rate between March 2, 2020 and June 16, 2020. Cases are defined as confirmed (PCR-RT) cases. Deaths include deaths of confirmed cases. Departments represent 32 departments plus 4 special districts (Santa Marta, Cartagena, Buenaventura and Barranquilla) and the capital district (Bogotá). All models represented are a fixed effects model with intercepts for department. All models control for the logarithm of the 7-day moving average of new active cases in each department, for second-degree polynomials of the percentage of cases detected through contact tracing, and for the corresponding lag on the mobility index in six different modes of mobility: retail/recreation activities, trips to grocery/pharmacy stores, parks, transit stations and workplace sites. The mobility index corresponds to the percentage change with regards to the baseline, which is the median value for the corresponding day of the week, during the 5-week period Jan 3-Feb 6, 2020. The coefficients presented represent the logarithm of the percentage of cases traced (7-day moving average). Stratification is done for 21, 28, 42 and 56 days in advance of the observed date.

Seemingly, in Table 3, we display the results from the random-effects design where the number of ICU beds per department were included with no major changes compared to the fixed-effects model and a range of reduction in mortality between 2.1% (model 1) and 3.4% (model 3) after a 10% increase on the proportion of cases detected through contact tracing. Importantly, these marginal effects are observed within the ranges of the levels of contact tracing identified. It is possible that these effects are not linear. At higher levels of contact tracing, there are either marginal decreasing returns or economies of scale that would modify the estimates.

**Table 3:** Random effects design at the department level on the effect of contact tracing on COVID-19 mortality.

Log of the number of COVID-19 deaths	Model 1 (lag for 21 days)			Model 2 (lags for 21 and 28 days)			Model 3 (lags for 21, 28, and 42 days)			Model 4 (lags for 21, 28, 42, and 56 days)		
	Effect	SE	*p*	Effect	SE	*p*	Effect	SE	*p*	Effect	SE	*p*
Logarithm of the percentage of cases traced 21 days before	-0.217	0.035	<0.001	-0.214	0.037	0.074	-0.229	0.041	<0.001	-0.203	0.042	<0.001
Logarithm of the percentage of cases traced 28 days before				-0.014	0.032	0.656	0.030	0.035	0.384	0.012	0.036	0.737
Logarithm of the percentage of cases traced 42 days before							-0.067	0.030	0.024	-0.062	0.030	0.040
Logarithm of the percentage of cases traced 56 days before										-0.080	0.026	0.002
Squared of the logarithm of the percentage of cases traced 21 days before	0.008	0.002	0.478	0.009	0.002	<0.001	0.010	0.002	<0.001	0.009	0.002	<0.001
Squared of the logarithm of the percentage of cases traced 28 days before				0.001	0.001	-0.001	0.001	0.002	0.327	-0.001	0.001	0.701
Squared of the logarithm of the percentage of cases traced 42 days before							0.001	0.001	0.205	0.002	0.001	0.058
Squared of the logarithm of the percentage of cases traced 56 days before										0.001	0.001	0.171
R-square (within)	0.503			0.521			0.468			0.449		
R-square (between)	0.519			0.543			0.677			0.747		
Observations (department/day units)	549			542			521			497		

Note: N = 54,931 confirmed cases reported to the National Institute of Health. The outcome is the logarithm of the daily number of COVID-19 deaths (7-day moving average) mortality rate between March 2, 2020 and June 16, 2020. Cases are defined as confirmed (PCR-RT) cases. Deaths include deaths of confirmed cases. Departments represent 32 departments plus 4 special districts (Santa Marta, Cartagena, Buenaventura and Barranquilla) and the capital district (Bogotá). All models represented are a fixed effects model with intercepts for department. All models control for the logarithm of the 7-day moving average of new active cases in each department, the logarithm of the number of ICU beds in that department as of June 15 to account for supply-side factors, for second-degree polynomials of the percentage of cases detected through contact tracing, and for the corresponding lag on the mobility index in six different modes of mobility: retail/recreation activities, trips to grocery/pharmacy stores, parks, transit stations and workplace sites. The mobility index corresponds to the percentage change with regards to the baseline, which is the median value for the corresponding day of the week, during the 5-week period Jan 3-Feb 6, 2020. The coefficients presented represent the logarithm of the percentage of cases traced (7-day moving average). Stratification is done for 21, 28, 42 and 56 days in advance of the observed date.

In Table 4, we present the results of the robustness checks including percentage of ICU occupation with a different set of cases, where an increase in 10% in the proportion of cases identified through contact tracing is related to mortality reductions in the next 21 to 28 days of between 0.8% and 2.2%, depending on the model.

**Table 4:** Fixed effects design at the department level on the effect of contact tracing on COVID-19 mortality.

Log of the number of COVID-19 deaths	Model 1 (lag for 21 days)			Model 2 (lags for 21 and 28 days)		
	Effect	SE	*p*	Effect	SE	*p*
Logarithm of the percentage of cases traced 21 days before	-0.078	0.034	0.024	-0.082	0.034	0.017
Logarithm of the percentage of cases traced 28 days before				-0.139	0.039	<0.001
Squared of the logarithm of the percentage of cases traced 21 days before	0.000	0.001	0.991	0.000	0.002	0.012
Squared of the logarithm of the percentage of cases traced 28 days before				0.004	0.019	0.170
R-square (within)	0.307			0.351		
R-square (between)	0.665			0.680		
Observations (department/day units)	383			383		

Note: N = 112,279 confirmed cases reported to the National Institute of Health between June 17, 2020 and July 15, 2020. The outcome is the logarithm of the daily number of COVID-19 deaths (7-day moving average) mortality rate between June 17, 2020 and July 15, 2020. Cases are defined as confirmed (PCR-RT) cases. Deaths include deaths of confirmed cases. Departments represent 26 departments plus 4 special districts (Santa Marta, Cartagena, Buenaventura and Barranquilla) and the capital district (Bogotá). We excluded the departments of Amazonas, Choco, Guaviare, Guainia, Vaupes and Vichada because they did not have consistent data on ICU occupation in the 28-day sub study period. All models represented are a fixed effects model with intercepts for department. All models control for the logarithm of the 7-day moving average of new active cases in each department, daily ICU occupation in that department as to account for supply-side factors, for second-degree polynomials of the percentage of cases detected through contact tracing, and for the corresponding lag on the mobility index in six different modes of mobility: retail/recreation activities, trips to grocery/pharmacy stores, parks, transit stations and workplace sites. ICU occupation for five dates (June 16, 19, 20, 22, and 25) in the 28-days of this sub study period were interpolated. The mobility index corresponds to the percentage change with regards to the baseline, which is the median value for the corresponding day of the week, during the 5-week period Jan 3-Feb 6, 2020. The coefficients presented represent the logarithm of the percentage of cases traced (7-day moving average). Stratification is done for 21 and 28 days in advance of the observed date.

## Conclusion

This study is the first to our knowledge using empirical data that identifies substantial mortality reductions associated to contact tracing for COVID-19. This paper also provides guidance on its effectiveness not only for Colombia, but also potentially for other LMICs. We found that an increase in 10% of cases detected through contact tracing in the 3 to 8 weeks before might be related to reductions in mortality ranging between 0.8% and 3.4%

Contact tracing is a known infection containment strategy recommended by the World Health Organization [22]. Modeling studies suggest that rapid identification, testing, and isolation of COVID-19 contacts increase detection of secondary cases and avoids the spread to tertiary cases, which might have an impact on mortality and ICU occupation. Further, contact tracing might allow for more distal effects, including easing lockdown restrictions [12, 26, 27].

Contact tracing capabilities often rely on robust public health infrastructure, well-trained human resources, and adequate funding, which in many LMIC’s might be lacking, especially in rural areas. One of the many challenges of contact tracing is keeping track of the contacts as the epidemic grows. The ability to trace enough contacts in a timely fashion [13] is substantially better when contact rates are lower. Previous modeling work suggests a robust synergic effect between contact rates and the ability of contact tracing, primarily mediated by the traceability of those [26].

Traditional strategies for contact tracing have always relied on trained staff, which many times include volunteers, students and retirees, as well as health professionals, NGO workers, and community members [8]. They carry out tracking activities that include locating and identifying infectious case contacts, and with coordinating support, allow for the monitoring and management of information to guide decision-making. Another strategy is community-based contact tracing which allows for improved community engagement and ownership of this epidemiological surveillance measures [28].

Finally, there are technology-based contact tracing strategies that take advantage of mobile phone apps to identify proximity of cases. From a public health perspective, these technologies are highly useful, but they often present challenges including privacy concerns and limitations on mobile network coverage [29].

Amid a pandemic and many competing demands, decision-makers struggling to contain the epidemic have faced challenges prioritizing contact tracing. There are several possible reasons for this. First, contact tracing is highly labor-intensive. It requires large numbers of skilled personnel to conduct parallel processes of both contact identification reported by detected cases and reaching out to reported contacts [7, 15]. Moreover, contact tracing needs an integrated information and communication system, streamlined processes so the tracing can be done within 72 hours, trustworthy local health institutions to which the population feels they can provide information on contacts, and buy-in from decision-makers and health institutions [16–19, 26, 30]. Third, contact tracing efficacy depends on the impact on isolation for those suspected and confirmed cases. Subsidies to support individuals on isolation represent a fiscal and logistic challenge that many LMIC are not yet equipped to provide at a large scale, particularly when cases are rapidly growing. Fourth, the fragmentation of the response across levels of government further increases the complexity of the response. In addition, competing policy instruments of infection control such as lockdowns were successful at the start of the pandemic but have proven to be less sustainable than expected, particularly in some settings in LMIC [11, 31–34]. However, in a context like this, decision-makers seem less likely to update previous beliefs as more information is available, and adopt new strategies for medium and long-term infection control [35]. These factors make it challenging to mobilize in such a short time, the human and fiscal constraints faced during this pandemic.

### Limitations

This paper presents some limitations. 1) We are using a performance parameter to proxy contact tracing, which might be endogenously related to non-pharmaceutical interventions’ performance. However, we control for mobility indices to reduce potential endogeneity due to concurrent lockdowns. 2) For our metric of contact tracing to capture mortality effects, it needs to be accompanied by the contacts’ isolation. As we cannot measure isolation performance from this data, the assumption is that there is homogeneous isolation compliance across departments. This is important because contact tracing is not effective without effective isolation, and our indicator might represent a broader indicator for epidemiological surveillance performance rather than only contact tracing. 3) Officially confirmed COVID deaths might be underestimated as some suspected cases might not be classified as COVID-19 deaths. A recent report of the Ministry of Health flagged this issue [36], but no specific figures on COVID-19 suspected mortality have been provided at this time. However, the ministry estimates overall excess mortality of only 3.8% for the first semester of 2020. 4) We are providing data only for Colombia because of the datasets’ quality that can be obtained. However, we expect this paper’s lessons to guide decision-makers in other LMICs about the impact of contact tracing on COVID-19 mortality. 5) As mentioned earlier, our findings are marginal effects observed within the ranges of contact tracing identified. As effects might not be linear, we might observe that at higher levels of contact tracing, there are either marginal decreasing returns or economies of scale that would modify the estimates. 6) As discussed in the methods section, we assume that the percentage of detected cases vs. undetected cases is constant in the study period. We indirectly tested this assumption assessing changes in the trends of detection for asymptomatic cases, implying this potential change should not be of concern on our measurement.

Our study suggests contact tracing is effective in reducing mortality. Nonetheless, no epidemiological surveillance strategy should be isolated from other measures. Whereas we measure a performance metric for contact tracing, other tools such as increased testing, isolation, and economic and social support for contacts must be part of an integrated approach.
